# Observations to Prove That the Hydrophobia Is Not, as Some Writers Have Asserted, Unknown in America. To Which Is Added, an Account of the Decussation of the Optic Nerves in Quadrupeds

**Published:** 1784

**Authors:** 


II. *Observations to prove that the Hydrophobia is not, as some writers have asserted, unknown in America. To which is added, an account of the decussation of the optic nerves in quadrupeds. Communicated in a letter to* Dr. Simmons, *F. R. S. by* Frederick Michaelis, *M. D. physician to the*

*Landgrave of Hesse Cassel, and late Physician General to the Hessian troops in America.*

IT is the opinion of many men of learning, both in Europe and America, that canine madness is a disorder unknown in the western world, and we find this idea in some of our principal writers on the natural history of America. One of them (Mr. Paw) even goes so far as to account for this surprizing difference, by saying, that the climate is too damp to admit of the disease; a kind of explanation, which, I confess, I do not understand.

Having always doubted the fact, I made it my business to investigate the matter; nor was it long before a decisive accident of the kind happened. A Hessian soldier, then a prisoner at Lancaster, was attacked with symptoms of hydrophobia after being bit by a mad dog, and the person, in whose house he lived, opened his veins, and suffered him to bleed to death.

A medical friend of mine at New York (Dr. Bard, senior) communicated to me two similar cases; the one of a person who died of the true hydrophobia, after being bit by a mad dog, and the other of a man who attempted to force medicines down the throat of a cow that had been bit bit by a mad dog; unluckily he happened to have a ſlight ſore on one of his fingers, which imbibed the poiſon, though he was not bit, and he died of the hydrophobia, though every thing was done that was thought likely to be of uſe.

Upon reading in the New-York paper, for July 1783, ſeveral inſtances of people's having died a little before in New England, with all the ſymptoms of hydrophobia, after having been bit by mad dogs; and obſerving ſoon after in the ſame paper, a very circumſtantial account of the caſe of a boy in Connecticut, who died of the hydrophobia eight weeks after being bit by a mad dog, I made it a point to inquire into the truth of theſe accounts, and every circumſtance was confirmed to me, by a friend who reſided in that quarter, and who added, that the dog laſt mentioned had bit a cow and a hog, who both died mad.

Nor are inſtances of this kind rare; Doctors Ruſh, Morgan, and Kuhn, three of the moſt diſtinguiſhed medical characters in America, have aſſured me, that they have now and then, in the courſe of their practice, met with inſtances of the true hydrophobia, occaſioned by the bites of mad dogs.

It

It has alſo been doubted, and is doubted even now by many of the inhabitants of the Weſt-India iſlands, whether the dogs there ever become mad, which is the more aſtoniſhing, as Hillary has mentioned his having ſeen the diſeaſe in Barbadoes.

---

*Of the decuſſation of the optic nerves in quadrupeds.*

